# Methylation of class II transactivator gene promoter IV is not associated with susceptibility to Multiple Sclerosis

**DOI:** 10.1186/1471-2350-9-63

**Published:** 2008-07-07

**Authors:** Sreeram V Ramagopalan, David A Dyment, Katie M Morrison, Blanca M Herrera, Gabriele C DeLuca, Matthew R Lincoln, Sarah M Orton, Lahiru Handunnetthi, Michael J Chao, A Dessa Sadovnick, George C Ebers

**Affiliations:** 1 Wellcome Trust Centre for Human Genetics, University of Oxford, Roosevelt Drive, Headington, Oxford, OX3 7BN, UK; 2 Department of Clinical Neurology, University of Oxford, Level 3, The West Wing, The John Radcliffe Hospital, Oxford, OX3 9DU, UK; 3 Department of Medical Genetics and Faculty of Medicine, Division of Neurology, University of British Columbia, G920, Detwiller Pavilion, VCHA – UBC Hospital, 2211 Wesbrook Mall, Vancouver, British Columbia, V6T 2B5, Canada

## Abstract

**Background:**

Multiple sclerosis (MS) is a complex trait in which alleles at or near the class II loci *HLA-DRB1 *and *HLA-DQB1 *contribute significantly to genetic risk. The MHC class II transactivator (*MHC2TA*) is the master controller of expression of class II genes, and methylation of the promoter of this gene has been previously been shown to alter its function. In this study we sought to assess whether or not methylation of the *MHC2TA *promoter pIV could contribute to MS disease aetiology.

**Methods:**

In DNA from peripheral blood mononuclear cells from a sample of 50 monozygotic disease discordant MS twins the *MHC2TA *promoter IV was sequenced and analysed by methylation specific PCR.

**Results:**

No methylation or sequence variation of the *MHC2TA *promoter pIV was found.

**Conclusion:**

The results of this study cannot support the notion that methylation of the pIV promoter of *MHC2TA *contributes to MS disease risk, although tissue and timing specific epigenetic modifications cannot be ruled out.

## Background

Genetic-epidemiological studies indicate unequivocally that there is a genetic influence on susceptibility to Multiple Sclerosis (MS) [1]. The only consistent genetic association with MS in Northern Europeans had been with extended MHC haplotypes especially those containing HLA-DRB1*1501 [1]. Recently, the interleukin 7 receptor (*IL7R*) and interleukin 2 receptor (*IL2R*) genes have been shown to be additional MS susceptibility loci [2,3]. However, any effect of *IL7R *or *IL2R *is small and it is clear that the MHC is the key MS susceptibility locus [4].

The MS MHC class II association has been fine mapped to the extended haplotype HLA-DQA1*0102-DQB1*0602-DRB1*1501-DRB5*0101 [5]. Intense linkage disequilibrium within the MHC has prevented the exact susceptibility locus from being conclusively identified. Analysis of the MHC region with a large number of markers as well as classical typing show evidence for the involvement of the class II region only [6,7]. However, the paradigm is more complex than one in which the HLA-DRB1*15 allele acts solely to increase MS risk. Our previous investigations have shown that HLA-DRB1*15 and HLA-DRB1*17 bearing haplotypes increase risk of MS, and HLA-DRB1*14 and HLA-DRB1*11 bearing haplotypes are protective [8,9]. Additionally, HLA-DRB1*10, DRB1*01 and DRB1*08 interact with HLA-DRB1*15 to influence disease risk [8,9].

Given the unequivocal MHC class II association with MS, the amount and cellular distribution of class II molecules may therefore be important factors in determining susceptibility to the disease. MHC class II molecule expression is regulated primarily through a transcriptional co-activator termed *MHC2TA *[10]. *MHC2TA *functions as a non-DNA-binding co-activator that coordinates multiple events that are required for the activation of transcription including the recruitment of transcription factors and phosphorylation of RNA Polymerase II [11]. The highly regulated pattern of expression of the gene encoding *MHC2TA *dictates where, when and to what level MHC class II genes are expressed [11]. Transcription of the gene encoding *MHC2TA *is controlled by a large regulatory region that contains three independent promoters (pI, pIII and pIV) [11]. The promoter pIV is essential for driving *MHC2TA *expression in cells that are sensitive to interferon-γ, and it has been shown that methylation of CpG dinucleotides in this promoter region can influence the expression of *MHC2TA *and thus MHC class II molecules [12].

Given a contentious association of *MHC2TA *polymorphisms with susceptibility to MS [13,14], we sought to assess whether or not methylation of the *MHC2TA *pIV promoter could contribute to MS aetiology using a cohort of monozygotic discordant twins, potentially ideal for entangling genetic and epigenetic contributions to disease susceptibility.

## Methods

### Subjects

All subjects used in the study were ascertained through the ongoing Canadian Collaborative Project on the Genetic Susceptibility to MS (CCPGSMS), for which the methodology has been previously described [15,16]. Each participating centre of the CCPGSMS obtained ethical approval (as set out in the Helsinki Declaration) from the relevant institutional review board, and the entire project was reviewed and approved by the University of British Columbia. Blood was obtained with appropriate consent.

Fifty pairs of monozygotic discordant twins (100 samples in total, 35 female and 15 male pairs) were chosen for analysis. The clinical data for the MS patients is shown in Table 1. The average age at blood sampling was 41.1 years (standard deviation = 3.7 years). 31 (62%) twin pairs were HLA-DRB1*15 positive.

**Table 1:** Clinical details of MS patients

**Clinical/demographic details**	
**Sample Size *(n)***	50
**Mean age of onset (years)**	31.1
**% Relapsing Remitting MS**	68

### CpG Dinucleotide Prediction

The sequence of the pIV promoter from the NCBI Build 36.1 reference sequence was analysed to identify CpG islands that could be methylated. The methodology for this is described in [17].

### Sequencing of promoter pIV

Total genomic DNA was extracted from whole blood as part of the CCPGSMS. PCR was performed using the primers shown in Table 2 under standard conditions [18] with an annealing temperature of 60 degrees Celsius and using AmpliTAQ gold (Applied Biosystems), yielding a PCR amplicon 257 base pairs in size. Sequencing reactions were carried out using BigDye v3.1 after which the DNA was sequenced using an ABI 3700 automated sequencer.

**Table 2:** Primer sequences used for sequencing

**Primers**	
**Forward Primer**	GGTTGGACTGAGTTGGAGAGA
**Reverse Primer**	GGAGCAACCAAGCACCTACT

### Bisulfite treatment and Methylation Specific PCR

Genomic DNA was treated using methylSEQr Bisulfite Conversion Kit from Applied BioSystems, following the manufacturer's protocol. This converts unmethylated cytosines to uracils and leaves methylated cytosines unchanged. Methylation specific PCR [19], using methylated DNA and unmethylated DNA specific primer sets was performed on treated DNA to detect methylation of the CpG island in the MHC2TA promoter. PCR was performed using the primers shown in Table 3 under standard conditions [18] with an annealing temperature of 55.5 degrees Celsius. Each PCR was performed twice for each sample to ensure validity of results. Universal methylated DNA, universal unmethylated DNA (both from CpGenome™) and water was used as positive, negative and blank controls respectively. Amplified fragments were confirmed by a 2.0% agarose gel.

**Table 3:** Primer sequences used for methylation specific PCR

**Primers**	**Sequence**	**Product Size**
**Methylated Forward**	TGTTTGGTTGTTTTATAGTTTGGTTC	60 bp
**Methylated Reverse**	CTACTAATAACCTCTCCCTCCCG	
**Unmethylated Forward**	TTGGTTGTTTTATAGTTTGGTTTGA	157 bp
**Unmethylated Reverse**	CTACTAATAACCTCTCCCTCCCAC	

## Results

**Figure 1:**
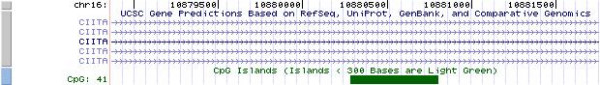
Predicted CpG island in the MHC2TA gene promoter IV.

In silico prediction of CpG islands in the pIV promoter uncovered 1 potential site (Figure 1) Sequencing of the region did not identify any polymorphisms in the pIV promoter sequence in any of the twin pairs.

**Figure 2:**
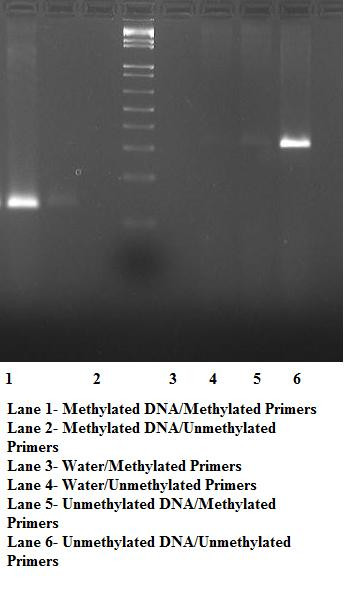
Gel electrophoresis of control and blank DNA amplicons after methylation specific PCR.

Methylation specific PCR was able to distinguish between methylated and unmethylated control samples (Figure 2). All twin DNA samples produced amplicons only with the unmethylated DNA specific primers.

## Discussion

Multiple sclerosis is unambiguously associated with the MHC class II region [6] and this locus exerts the strongest genetic effect on the risk of developing the disease [4]. *MHC2TA *is the master regulator of MHC class II gene expression and therefore variability at the *MHC2TA *gene could conceivably influence susceptibility to MS.

In this investigation we studied the sequence variability of the pIV promoter of the *MHC2TA *gene and found no variation. This is in agreement with previous studies and this conservation may be a result of the importance of this promoter to gene function.

The only known epigenetic modification of DNA in mammals is methylation of cytosine at position C5 in CpG dinucleotides [20]. DNA methylation affects transcription directly, by influencing the binding of specific transcription factors, and indirectly, by recruiting methyl-CpG-binding proteins and their associated chromatin remodeling activities. It has been shown that methylation of the pIV promoter can influence *MHC2TA *expression. Monozygotic twins share a common genotype. However, genetically identical twin pairs exhibit differences in susceptibility to many diseases, including MS, where the monozygotic twin concordance rate at its highest does not exceed 30% [21]. There are several possible explanations for these observations, one of these being the existence of epigenetic differences. In this study, we used a cohort of monozygotic MS discordant twins to examine whether methylation differences of the *MHC2TA *promoter could explain differences in susceptibility to disease. We did not detect methylation of CpG dinucleotides in the pIV promoter in any of our samples, either MS affected or not. Although this study argues against a role of methylation of *MHC2TA *in MS disease pathogenesis, it must be remembered that whilst genomic information is uniform among the different cells of a complex organism, the epigenome varies from tissue to tissue, controlling the differential expression of genes and providing specific identity to each cell type. Hence, by looking solely at peripheral blood mononuclear cells we may have missed tissue specific methylation of the *MHC2TA *promoter. Furthermore, a recent study which compared global and locus specific methylation patterns in monozygotic twins, showed that although indistinguishable in early life, epigenetic profiles of monozygotic twins change with age [22] and hence for an adult onset disease with susceptibility determined early in life [23,24] timing of any epigenetic changes may be crucial, and our study may not have been able to detect methylation of *MHC2TA *at an early age that has since decayed. Additionally, we may have missed low level methylation patterns and it would be necessary to examine every CpG dinucleotide of *MHC2TA *to be confident that an association between methylation and disease had not been missed just because the wrong markers had been typed.

## Conclusion

In summary, although our results do not completely rule out the possibility of an association between methylation of *MHC2TA *and MS we believe that our data is sufficient to exclude a *major *effect of methylation of this gene in MS pathology.

## Competing interests

The authors declare that they have no competing interests.

## Authors' contributions

GCE conceived and designed the experiments. SVR, DAD, BMH, GCD, MRL, SMO, LH, MJC and ADS performed the experiments. SVR and GCE analyzed the data and wrote the paper.

## Pre-publication history

The pre-publication history for this paper can be accessed here:


